# Profile of Lipoprotein Subclasses in Chinese Primary Open-Angle Glaucoma Patients

**DOI:** 10.3390/ijms25084544

**Published:** 2024-04-21

**Authors:** Changzhen Fu, Jianming Xu, Shao-Lang Chen, Chong-Bo Chen, Jia-Jian Liang, Zibo Liu, Chukai Huang, Zhenggen Wu, Tsz Kin Ng, Mingzhi Zhang, Qingping Liu

**Affiliations:** 1 Joint Shantou International Eye Center of Shantou University and The Chinese University of Hong Kong, Shantou 515041, China; fcz@jsiec.org (C.F.); xjm@jsiec.org (J.X.); csl@jsiec.org (S.-L.C.); ccb@jsiec.org (C.-B.C.); ljj@jsiec.org (J.-J.L.); liuzibo0910@gmail.com (Z.L.); hck@jsiec.org (C.H.); wzg@jsiec.org (Z.W.); micntk@hotmail.com (T.K.N.); 2 Department of Ophthalmology and Visual Sciences, The Chinese University of Hong Kong, Hong Kong, China

**Keywords:** POAG, lipoprotein subclasses, small dense LDL, oxidized LDL

## Abstract

To investigate the plasma lipoprotein subclasses in patients with primary open-angle glaucoma (POAG), a total of 20 Chinese POAG patients on intraocular pressure (IOP)-lowering treatment and 20 age-matched control subjects were recruited. Based on the levels of total cholesterol (TC) and low-density lipoprotein cholesterol (LDL-C), the study subjects were divided into elevated- and normal-level subgroups. The plasma lipoprotein, lipoprotein subclasses, and oxidized LDL (oxLDL) levels were quantitatively measured. The discrimination potential of the lipoproteins was evaluated using the area under the receiver operating characteristic curve (AUC), and their correlation with clinical parameters was also evaluated. Compared to the control subjects with elevated TC and/or LDL-C levels, the levels of TC, LDL-C, non-high-density lipoprotein cholesterol (non-HDL), LDL subclass LDL3 and small dense LDL (sdLDL), and oxLDL were significantly higher in POAG patients with elevated TC and/or LDL-C levels. No differences in any lipoproteins or the subclasses were found between the POAG patients and control subjects with normal TC and LDL-C levels. Moderate-to-good performance of TC, LDL-C, non-HDL, LDL3, sdLDL, and oxLDL was found in discriminating between the POAG patients and control subjects with elevated TC and/or LDL-C levels (AUC: 0.710–0.950). Significant negative correlations between LDL3 and sdLDL with retinal nerve fiber layer (RNFL) thickness in the superior quadrant and between LDL3 and average RNFL thickness were observed in POAG patients with elevated TC and/or LDL-C levels. This study revealed a significant elevation of plasma lipoproteins, especially the LDL subclasses, in POAG patients with elevated TC and/or LDL-C levels, providing insights on monitoring specific lipoproteins in POAG patients with elevated TC and/or LDL-C.

## 1. Introduction

Glaucoma, a leading cause of irreversible blindness and visual impairment, is characterized by the progressive degeneration of retinal ganglion cells (RGCs) and the thinning of the retinal nerve fiber layer (RNFL), resulting in gradual visual field loss [1,2,3]. Reported risk factors for glaucoma, based on clinical trials, include elevated intraocular pressure (IOP), advanced age, race, myopia, optic nerve susceptibility, and positive family history [1,2]. Primary open-angle glaucoma (POAG) is the most prevalent form of glaucoma, and IOP is the only clinically recognized modifiable risk factor for POAG progression. In spite of the pharmacological or surgical interventions to lower the IOP, some patients still experience a progressive loss of RGCs and visual field [4,5]. Even among well-managed patients, approximately 15% will become blind in at least one eye in 20 years [6,7]. As POAG is a multifactorial and heterogeneous disease, it is warranted to further investigate the etiology of POAG beyond the elevated IOP.

We previously identified the caveolin-1 (*CAV1*) and ATP-binding cassette A1 (*ABCA1*) genes associated with POAG in genome-wide association studies [8,9]. Both CAV1 and ABCA1 are crucial regulators in lipid homeostasis, in that ABCA1 is involved in high-density lipoprotein (HDL) biogenesis and facilitates cholesterol efflux [10], whereas CAV1 is involved in cellular cholesterol absorption and cellular cholesterol efflux by interacting with ABCA1, scavenger receptor class I (SR-BI), and CD36 [11,12]. Our previous study demonstrated that POAG patients had significantly higher fasting plasma triglycerides (TGs) but lower high density lipoprotein cholesterol (HDL-C) levels at recruitment as compared to the control subjects [8]. Other studies reported a higher prevalence of elevated total cholesterol (TC) and low-density lipoprotein cholesterol (LDL-C) levels in POAG patients [13], and the risk of POAG is positively associated with elevated TC levels, while the treatment to reduce TC might decrease the risk of POAG [14,15]. In contrast, a negative correlation between HDL-C levels and the risk of POAG [16], an inverse association between hyperlipidemia and POAG, as well as a lack of correlation between the blood levels of TC, LDL-C, and HDL-C with POAG were also reported [16,17,18,19]. For the lipoprotein subclasses, HDL3 was found to be significantly associated with POAG [19]. Lipoprotein subclasses are heterogeneous particles derived from plasma lipoproteins, such as LDL and HDL, which can be further sub-classified based on their sizes and densities [20]. LDL, especially the small granular subclasses, is susceptible to oxidation, resulting in the formation of oxidized LDL (oxLDL) [21,22]. Cholesterol, phospholipids, and triglycerides are the main lipid components of the lipoprotein subclasses and oxLDL, and they exhibit significant variations in terms of sizes, densities, physicochemical compositions, and electric charges. The differences not only contribute to their heterogeneity in origin, metabolic behavior, tissue distribution, vascular wall permeability, oxidation propensity, and antioxidant activity [23,24,25,26], but also shape their unique functions that play diverse roles in disease pathogenesis and progression [27,28]. However, the specific functions are often masked by broad conventional lipid profiles. Moreover, the existing literature on the association between lipid disorders and POAG is limited due to inadequate adjustment for important potential confounders such as diabetes, disease status, use of cardiovascular medications such as statins, hypertension, immune system disorders, etc., all of which may have potential associations with IOP that could influence the association between POAG and blood lipoproteins. Therefore, an in-depth analysis on the associations between lipoprotein subclasses and oxLDL with POAG is warranted. Here, we aimed to delineate the profile of lipoproteins and the subclass in the plasma samples of POAG patients. The discrimination potential of the lipoprotein subclasses and their correlation with clinical parameters were also evaluated.

## 2. Results

### 2.1. Demographic and Clinical Features of Study Subjects

The 20 POAG patients and 20 control subjects were subdivided into four groups based on their TC and LDL levels, with 10 subjects in each group. The ages of the study subjects ranged from 50 to 84 years old. A significant increase in cup-to-disc (C/D) ratio was observed between the POAG and control subjects in both elevated and normal TC and LDL-C level subgroups (*p* < 0.001) (Table 1). Yet, no significant differences were found between the POAG patients and control subjects, as well as the subgroups, in all other parameters.

### 2.2. Profiles of Lipoproteins in Primary Open-Angle Glaucoma Patients

The POAG patients with elevated TC and/or LDL-C levels showed significant increases in 13 lipoprotein parameters, including TC, LDL-C, oxLDL, apolipoprotein B (ApoB), ApoB/apolipoprotein AI (ApoAI), intermediate-density lipoprotein (IDL), IDL-C, LDL3, small density lipoprotein (sdLDL), very-low-density lipoprotein (VLDL), non-high-density lipoprotein cholesterol (non-HDL), TC/HDL-C ratio, and oxLDL/LDL-C ratio as compared to the POAG patients with normal TC and LDL-C levels (Table S1). Similarly, the control subjects with elevated TC and/or LDL-C levels showed significant increases in 10 lipoprotein parameters, including TC, LDL-C, TG, ApoB, IDL, IDL-C, LDL3, VLDL, non-HDL, and oxLDL/LDL-C ratio as compared to the control subjects with normal TC and LDL-C levels (Table S1). Elevations in ApoB, IDL-C, LDL3, VLDL, non-HDL, and oxLDL/LDL-C ratio were commonly found in the POAG patients and control subjects with elevated levels of TC and/or LDL-C. These indicated that the lipoprotein parameters could be altered in the subjects with elevated TC and/or LDL-C levels.

### 2.3. Subclasses of Lipoproteins in Primary Open-Angle Glaucoma Patients

**Figure 1:**
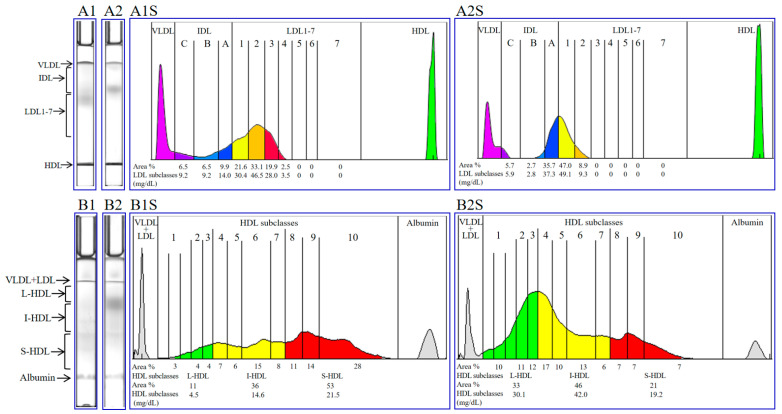
Representative images of electrophoretic migration and corresponding density scanning maps for LDL and HDL. The gel electrophoresis migration and density scanning maps of LDL (A1, A1S) and HDL (B1, B1S) profiles for a patient with POAG, as well as the LDL (A2, A2S) and HDL (B2, B2S) profiles for a control subject. VLDL, very-low-density lipoprotein; IDL, intermediate-density lipoprotein; LDL, low-density lipoprotein; HDL, high-density lipoprotein; L-HDL, large high-density lipoprotein; I-HDL, intermediate HDL; S-HDL, small HDL.

The gel electrophoresis migration and corresponding density scanning maps of LDL (Figure 1, A1, A1S, LDL: 140.8 mg/dL) and HDL (Figure 1, B1, B1S, HDL: 40.6 mg/dL) for a patient with POAG are presented. Similarly, the gel electrophoresis migration and corresponding density scanning maps of LDL (Figure 1, A2, A2S, LDL: 104.4 mg/dL) and HDL (Figure 1, B2, B2S, HDL: 91.3 mg/dL) for a control subject are also shown. The concentration of each LDL or HDL lipoprotein subclass is indicated at the bottom of the scan maps.

A significant increase in TC (POAG: 5.45 ± 1.11 mmol/L; control: 4.77 ± 0.80 mmol/L, *p* < 0.05), non-HDL (POAG: 4.07 ± 1.07 mmol/L; control: 3.36 ± 0.68 mmol/L, *p* < 0.05) and LDL3 (POAG: 22.54 ± 11.12 mg/dL; control: 15.14 ± 8.98 mg/dL, *p* < 0.05) was found between the POAG and control subjects. In the POAG patients with elevated TC and/or LDL-C levels, significant increases in TC, LDL-C, non-HDL, LDL3, sdLDL, and oxLDL levels were found as compared to control subjects with elevated TC and/or LDL-C levels (Table 2). No significant differences were found in any lipoprotein parameters between the POAG patients and control subjects with normal TC and LDL-C levels. Our results suggested that the POAG patients with elevated TC and/or LDL-C levels could have higher levels of lipoproteins.

### 2.4. Receiver Operating Characteristic (ROC) and Clinical Correlation Analyses

**Figure 2:**
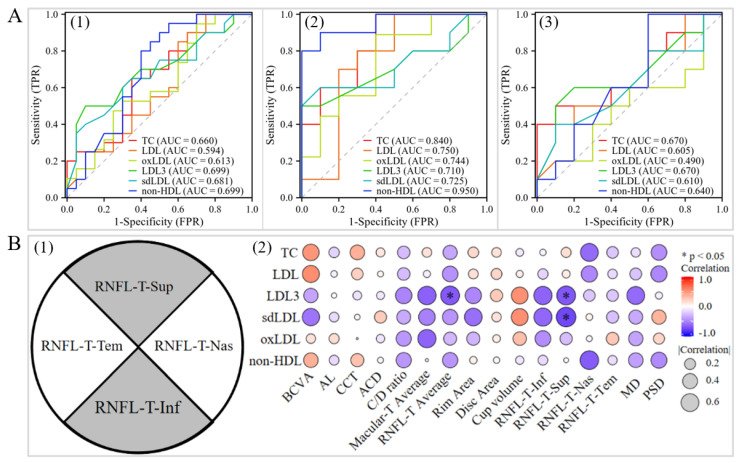
ROC curve and clinical correlation analysis. (**A**) The discrimination of TC, LDL-C, non-HDL, LDL3, sdLDL, and oxLDL between POAG patients and control subjects (**1**); POAG patients and control subjects in both elevated TC and/or LDL-C levels (**2**); and POAG patients and control subjects in both normal TC and LDL-C levels (**3**). (**B**) Pattern maps of the superior, inferior, nasal, and temporal quadrant RNFL layers (**1**) and heat maps illustrating the correlation between the lipoprotein parameters and clinical parameters (**2**). BCVA, best-corrected visual acuity; AL, axial eye length; CCT, central corneal thickness; C/D ratio, cup-to-disc ratio; Macular-T, macular thickness; RNFL-T, retinal nerve fiber layer thickness; CV, cup volume; Inf, inferior quadrant; sup; superior quadrant; Nas, nasal quadrant; Tem, temporal quadrant; MD, mean deviation; PSD, pattern standard deviation. Data are presented as mean ± standard deviation (SD), * *p* < 0.05.

The ROC analysis showed that TC, LDL-C, non-HDL, LDL3, sdLDL, and oxLDL showed moderate-to-good discrimination between the POAG patients with elevated TC and/or LDL-C levels and the control subjects with elevated TC and/or LDL-C levels. The area under the ROC curve (AUC) was from 0.710 to 0.950 (AUC: 0.710–0.950). In contrast, the AUC was not satisfactory in the comparison between the POAG patients with normal TC and LDL-C levels and the control subjects with normal TC and LDL-C levels (AUC < 0.7, Figure 2A).

Spearman correlation of TC, LDL-C, oxLDL, sdLDL, and non-HDL with 16 clinical variables was conducted in the POAG patients with elevated TC and/or LDL-C levels. The results showed that LDL3 and sdLDL were negatively associated with superior RNFL thickness, while LDL3 was negatively correlated with average RNFL thickness (Figure 2B).

## 3. Discussion

In this study, we investigated the lipoproteins and their subclasses in the plasma samples of POAG patients under IOP-lowering treatment. Our results revealed a significant increase in TC, LDL-C, non-HDL, LDL3, sdLDL, and oxLDL in the POAG patients with elevated TC and/or LDL-C levels as compared to the control subjects with elevated TC and/or LDL-C levels. The TC, LDL-C, non-HDL, LDL3, sdLDL, and oxLDL showed moderate-to-good discrimination between the POAG patients with elevated TC and/or LDL-C levels and the control subjects with elevated TC and/or LDL-C levels. Moreover, negative correlation was observed between the LDL3 and sdLDL levels and superior quadrant RNFL thickness, as well as between the LDL3 levels with average RNFL thickness. Our results suggested that elevated lipoprotein levels, including the conventional lipoproteins, oxLDL, and LDL subclass, were seen in the POAG patients with elevated TC and/or LDL-C levels.

Plasma TC and LDL are considered as traditional lipid risk factors [29,30,31,32,33]. Previous studies have demonstrated that the metabolic characteristics of lipoprotein subclasses and modifications are influenced by the types and conditions of the diseases and by TC and LDL levels [34,35,36]. In this study, eight common lipoprotein parameters were found to be elevated in the study subjects with elevated TC and/or LDL-C levels, both in the POAG patients and in the control subjects. In view of this, we conducted stratified analyses based on the TC and LDL levels to avoid potential bias in the correlation analysis between lipoprotein profiles and POAG.

Controversial results in the association between the conventional lipid parameters and POAG have been reported [13,14,15,16,17,18]. In this study, we observed a significant increase in the small granular subclasses of LDL (LDL3 and sdLDL) and the oxidized derivative oxLDL in POAG patients with elevated TC and/or LDL-C levels. These lipoproteins have been considered as key regulators in atherosclerotic cardiovascular disease and other vascular-related diseases [37,38]. sdLDL, encompassing LDL3 to LDL7, refers to the smaller and denser subclasses of LDL. These subclasses are susceptible to oxidative modification and glycosylation, and show reduced affinity to LDL receptors and increased binding to proteoglycans, which could result in delayed plasma clearance, facile adhesion to the vascular wall, and enhanced penetration into the vascular wall [39,40,41,42]. These can cause atherosclerosis in large vessels as well as the atherosclerosis and occlusion of small vessels [43], which lead to a diminished blood vessel diameter and reduced blood flow. In glaucoma, multiple studies consistently demonstrated a reduction in ocular blood flow (OBF), especially in normal-tension glaucoma patients [44,45,46]. In this study, we revealed significantly elevated levels of both LDL3 and sdLDL in POAG patients with elevated TC and/or LDL-C levels, which were negatively correlated with superior quadrant RNFL thickness and average RNFL thickness (Figure 2). The RNFL thinning in the POAG patients with elevated TC and/or LDL-C levels may be related to reduced blood supply due to the increase in blood LDL3 and sdLDL. Both LDL3 and sdLDL are highly susceptible to oxidation, leading to their transformation into oxLDL [47,48]. oxLDL can trigger oxidative stress responses along with inflammatory reactions and macrophage foam formation [49,50,51]. In addition to age-related factors, oxidative stress and inflammation are also involved in POAG [52]. A previous study reported elevated levels of oxLDL in conjunctival stromal cells and in the extracellular matrix of POAG patients [53]. Moreover, reduced antioxidant levels and increased markers of oxidative stress were also found in the aqueous humor of POAG patients [54,55,56]. These suggest a possible correlation between oxLDL and POAG. In this study, we observed significantly elevated levels of oxLDL in blood samples of the POAG patients with elevated TC and/or LDL-C levels (Table 2), further indicating the contribution of oxLDL in POAG. Therefore, LDL3, sdLDL, and oxLDL may be present in POAG patients with elevated TC and/or LDL levels.

HDL and LDL exhibit similar structural characteristics and surface chemistry but play different physiological roles. HDL promotes cholesterol transport back to the liver for excretion, especially the HDL3 subclasses, and it promotes cholesterol efflux via ABCA1. HDL has been shown to possess antioxidant, anti-inflammatory, and anti-thrombotic activities that can confer protection to the vascular endothelium [57,58,59,60]. In POAG, it has been reported that higher levels of HDL3 in the blood are associated with a lower risk of POAG [19]. However, in this study, no significant correlation was found between HDL3 and POAG or the subgroups (Table 2). This discrepancy may be attributed to different populations. In addition, the functional performance of HDL exhibits dynamic changes dependent on environmental factors such as its anti-inflammatory properties, anti-oxidation capacity, and capability to reverse cholesterol transport (RCT) [61]. Measuring the blood HDL-C parameter alone cannot accurately reflect the functionality of HDL particles [62,63]. Therefore, deep phenotyping should be conducted to elucidate the relationship between specific functional components within HDL particles in POAG patients.

There were several limitations in this study. First, this study lacks long-term lipoprotein monitoring during disease progression. Second, the sample size of this study is small, which reduces the statistical power of the analyses. Third, to reduce the influence of blood pathological TC and LDL-C levels on lipoprotein subclasses and oxLDL, the POAG and control subjects were divided into two subgroups, each based on the levels of TC and LDL-C. Our results identified a significant increase in TC and LDL-C in the POAG patients with elevated TC and/or LDL-C levels as compared to the control subjects with elevated TC and/or LDL-C levels. It is important to consider the high prevalence and positive correlation of elevated cholesterol levels with POAG, as well as the potential benefits of cholesterol-lowering treatments [13,14,15,16,17,18,19]. Therefore, POAG patients with elevated TC and/or LDL-C levels may exhibit even higher levels of TC and/or LDL-C as compared to control subjects with elevated TC and/or LDL-C levels. However, this limitation should not be considered as the main factor influencing the bias of the results. Fourth, the confounding factors, including disease duration and diet information, were not considered in the analyses.

In summary, this study revealed that the POAG patients with elevated TC and/or LDL-C levels showed higher TC, LDL-C, non-HDL, LDL3, sdLDL, and oxLDL levels as compared to the control subjects with elevated TC and/or LDL-C levels. LDL3 and sdLDL were also associated with RNFL thinning. This study provides insight, considering lipoproteins and a subclass of LDL in POAG patients with elevated TC and/or LDL-C levels.

## 4. Materials and Methods

### 4.1. Study Subjects

This study was approved by the Human Medical Research Ethics Committee of the Joint Shantou International Eye Center (JSIEC) of Shantou University and the Chinese University of Hong Kong (approval number: EC20210803(6)-P03), and it is in accordance with the tenets of the Declaration of Helsinki. Written informed consent was obtained from all study subjects prior to inclusion in this study after an explanation of the nature and possible consequences of the study. A total of 420 patients with POAG who underwent initial diagnosis and/or treatment monitoring at JSIEC between October 2021 and October 2022 were included in this study. POAG patients were diagnosed based on the following criteria: (1) presence of an open anterior chamber angle, with Shaffer Grade 2 or above in dark room gonioscopy without indentation; (2) IOP > 21 mmHg at the time of diagnosis; (3) evidence of characteristic glaucomatous optic disc changes, including narrowing of the neuroretinal rim or thinning of the retinal nerve fiber layer detected by optical coherence tomography (OCT); and (4) fulfilling Anderson’s criteria for minimal abnormality in a glaucomatous visual field [64]. In order to accurately assess the association between blood lipoproteins with POAG, we implemented a stringent overall exclusion criteria for confounding factors in POAG patients by (1) excluding POAG patients with other ocular diseases such as secondary glaucoma, exfoliation glaucoma, neovascular glaucoma, steroid-induced glaucoma, a history of previous glaucoma surgery or ocular trauma, etc., except for mild senile cataracts or refractive errors; (2) excluding POAG patients younger than 40 years old, and who have a history of diabetes, hypertension, autoimmune disorders, and cardiovascular disease (CVD), or who use cholesterol-lowering drugs (such as statins); (3) excluding POAG patients whose IOP did not decrease to <21 mmHg after treatment with 1–3 IOP-lowering medications, including rinzolamide, brinzolamide and timolol, carteolol hydrochloride, brimonidine tartrate, tafluprost and latanoprost, or those lacking complete clinical data. Finally, 21 POAG patients were screened, including 10 POAG patients with elevated TC and/or LDL-C, and 11 POAG patients with normal TC and LDL-C levels. We selected the only 10 POAG patients with elevated TC and/or LDL-C along with a matched number of 10 out of the remaining 11 POAG patients with normal TC and LDL-C levels for an analysis of plasma lipoproteins. Moreover, we included 10 age- and sex-matched control subjects with elevated TC and/or LDL-C, as well as another set of 10 age- and sex-matched control subjects with normal TC and LDL-C levels but that did not have any ocular diseases except mild senile cataracts or refractive errors.

### 4.2. Ophthalmic Examinations

All study subjects received comprehensive ophthalmic examinations, including best-corrected visual acuity (BCVA; logMAR scale), IOP measured by a Goldmann applanation tonometer (Haag-Streit, Koeniz, Switzerland), anterior chamber examined by slit-lamp biomicroscopy (Haag-Streit BQ-900; Koeniz, Switzerland), axial eye length (AL), central corneal thickness (CCT), and anterior chamber depth (ACD) measured by IOL Master (Carl Zeiss Meditec, Jena, Germany), visual fields defect (mean deviation (MD) and pattern standard deviation (PSD)) assessed by Humphrey MATRIX perimetry analyzer (Carl Zeiss Meditec, Jena, Germany), and peripapillary area, disc area, average macular thickness, and RNFL thickness quantified using Cirrus HD-OCT 4000 (Carl Zeiss Meditec, Jena, Germany).

### 4.3. Blood Collection and Lipoprotein Analysis

Fasting peripheral venous blood samples were collected in EDTA tubes. The plasma was isolated after centrifugation at 3000 rpm at 4 °C for 20 min, snap frozen, and stored at −80 °C before use.

The plasma biochemical molecules, including TC, LDL-C, HDL-C, TG, glucose, C-reactive protein (CRP), lipoprotein α (Lpα), and apolipoprotein AI (ApoAI), were measured using an automated biochemical analyzer. Human plasma oxLDL concentrations were measured using a Mercodia oxLDL ELISA Kit (Mercodia, Uppsala, Sweden), according to the manufacturer’s instructions.

The plasma samples were analyzed using the Lipoprint LDL System and Lipoprint HDL System (Quantimetrix Inc., Redondo Beach, CA, USA) by non-denaturing polyacrylamide gel electrophoresis. The LDL and HDL subclasses were quantified based on the measurements of LDL-C and HDL-C levels, as well as gel tube scans [65,66]. LDL subclasses included IDL-C, IDL-B, IDL-A, and LDL1-LDL7. The combined quantification of LDL1 and LDL2 was considered as large LDL, while the cumulative quantification of LDL3 to LDL7 was referred to as small dense LDL (sdLDL) [67]. The quantification of each LDL subclass was determined by multiplying the total LDL quantification of the plasma sample by the corresponding AUC. HDL subclasses, including HDL1-HDL10, were further categorized into three subclasses: large HDL (HDL1-HDL3), intermediate HDL (HDL4-HDL7), and small HDL (HDL8-HDL10) [68]. The quantification of each HDL subclass was determined by multiplying the total HDL quantification of the plasma sample by the corresponding AUC.

The units for TC, LDL-C, and HDL-C were converted from mmol/L to mg/dL with the formula mg/dL × 0.02586 = mmol/L. The non-HDL (mmol/L), which contains cholesterol in LDL, VLDL, IDL, Lpα, and all other lipoproteins [69,70], was calculated as TC (mmol/L) minus HDL-C (mmol/L) [71]. The lipoprotein ratios were calculated as oxLDL/HDL-C ratio = oxLDL (U/L)/HDL-C (mmol/L) ratio; oxLDL/LDL-C ratio = oxLDL (U/L)/LDL-C (mmol/L) ratio; and oxLDL/TC ratio = oxLDL (U/L)/TC (mmol/L) ratio [72,73].

### 4.4. Statistical Analysis

Based on the levels of TC and LDL-C, the POAG patients and control subjects were divided into 2 subgroups each (a total of 4 groups): (1) POAG patients or control subjects with elevated levels of TC and/or LDL-C (TC ≥ 5.2 mmol/L and/or LDL-C ≥ 3.4 mmol/L [74]), and (2) POAG patients or control subjects with normal levels of TC and LDL (TC < 5.2 mmol/L and LDL-C < 3.4 mmol/L [74]).

Statistical analyses and visualizations were obtained using the Xiantao Academic Tools (https://www.xiantaozi.com (accessed on 2023)) or spss 22.0 software (SPSS 22.0, Inc., Chicago, IL, USA). The categorical data were analyzed using the χ^2^ or Fisher’s exact test. The normal distribution of the data was analyzed using the Shapiro-Wilk normality test, and Levene’s test was used to evaluate the homogeneity of variance. Student *t* test, Welch *t* tests, and the Wilcoxon rank sum test were used to analyze the quantitative data. The data were presented as mean ± standard deviation (SD). *p* < 0.05 was considered as statistically significant.

## Figures and Tables

**Table 1:** Demographic and clinical features of study subjects.

Variables	Elevated TC and/or LDL-C			Normal TC and LDL-C		
	POAG (*n* = 10)	Control (*n* = 10)	*p* Value	POAG (*n* = 10)	Control (*n* = 10)	*p* Value
Age (years)	68.60 ± 7.12	65.30 ± 7.51	0.764 ^a^	68.40 ± 8.10	68.40 ± 9.20	1.000 ^a^
Male/female sex	7/3	5/5	0.361 ^b^	7/3	6/4	0.639 ^b^
Right IOP (mmHg)	14.30 ± 3.89	15.80 ± 3.91	0.401 ^a^	15.50 ± 5.32	12.70 ± 2.75	0.162 ^c^
Left IOP (mmHg)	14.00 ± 3.46	15.10 ± 3.04	0.460 ^a^	16.10 ± 4.04	12.90 ± 2.69	0.052 ^a^
Mean IOP (mmHg)	14.15 ± 2.97	15.45 ± 3.18	0.357 ^a^	15.80 ± 3.99	12.8 ± 2.37	0.060 ^c^
BMI (kg/m^2^)	24.43 ± 2.47	22.64 ± 3.41	0.215 ^a^	23.97 ± 3.70	23.02 ± 3.24	0.559 ^a^
Glucose (mmol/L)	6.82 ± 2.05	6.45 ± 1.16	0.971 ^d^	6.77 ± 1.54	6.20 ± 1.69	0.315 ^d^
CRP (mg/L)	1.12 ± 1.50	2.00 ± 1.06	0.055 ^d^	1.86 ± 2.79	0.73 ± 0.41	1.000 ^d^
SBP (mmHg)	137.50 ± 18.35	128.40 ± 11.40	0.120 ^a^	138.90 ± 14.45	138.10 ± 9.77	0.886 ^a^
DBP (mmHg)	81.00 ± 13.01	79.90 ± 9.42	0.831 ^a^	78.80 ± 6.46	82.70 ± 6.07	0.181 ^a^
BCVA (logMAR)	0.53 ± 0.34	0.80 ± 0.55	0.120 ^d^	0.49 ± 0.29	0.60 ± 0.28	0.396 ^a^
AL (mm)	23.26 ± 0.79	23.53 ± 0.51	0.377 ^a^	23.20 ± 0.84	23.73 ± 0.87	0.280 ^d^
CCT (µm)	549.30 ± 27.86	539.10 ± 35.16	0.481 ^a^	548.95 ± 55.88	526.60 ± 25.45	0.273 ^d^
ACD (mm)	3.35 ± 0.22	3.08 ± 0.44	0.010 ^a^	3.04 ± 0.17	3.23 ± 0.42	0.219 ^c^
C/D ratio	0.87 ± 0.05	0.32 ± 0.04	<0.001 ^d^	0.88 ± 0.04	0.32 ± 0.04	<0.001 ^d^

Elevated TC and/or LDL, POAG, or control subjects plasma TC ≥ 5.2 mmol/L and/or LDL-C ≥ 3.4 mmol/L; normal TC and LDL, POAG, or control subjects plasma TC < 5.2 mmol/L and LDL-C < 3.4 mmol/L; IOP, intraocular pressure; BMI, body mass index; CRP, C-reactive protein; SBP, systolic blood pressure; DBP, diastolic blood pressure; BCVA, best-corrected visual acuity; AL, axial length; CCT, central corneal thickness; ACD, anterior chamber depth; C/D ratio, cup-to-disc ratio. ^a^ Student *t* test; ^b^ Fisher’s exact test, ^c^ Welch *t* tests; ^d^ Wilcoxon rank sum test. Data are presented as mean ± standard deviation (SD).

**Table 2:** Subclasses of lipoproteins in primary open-angle glaucoma patients.

Variables	Elevated TC and/or LDL-C			Normal TC and LDL-C		
	POAG (*n* = 10)	Control (*n* = 10)	*p* Value	POAG (*n* = 10)	Control (*n* = 10)	*p* Value
TC (mmol/L)	6.31 ± 0.88	5.30 ± 0.40	0.006 ^c^	4.58 ± 0.42	4.16 ± 0.67	0.112 ^a^
LDL-C (mmol/L)	4.26 ± 0.90	3.53 ± 0.36	0.035 ^c^	2.66 ± 0.46	2.50 ± 0.53	0.483 ^a^
HDL-C (mmol/L)	1.39 ± 0.33	1.46 ± 0.43	0.688 ^a^	1.37 ± 0.25	1.28 ± 0.34	0.476 ^a^
TG (mmol/L)	2.08 ± 0.89	1.61 ± 0.74	0.222 ^a^	1.39 ± 0.64	1.07 ± 0.14	0.144 ^c^
oxLDL (U/L)	62.53 ± 14.31	50.05 ± 9.22	0.040 ^a^	49.03 ± 12.32	46.89 ± 9.79	0.672 ^a^
Lpα (mg/L)	133.90 ± 93.34	181.70 ± 145.34	0.393 ^a^	141.20 ± 127.41	95.30 ± 86.28	0.315 ^d^
ApoB (g/L)	1.10 ± 0.22	0.95 ± 0.13	0.064 ^a^	0.75 ± 0.16	0.75 ± 0.09	0.987 ^a^
ApoAI (g/L)	1.38 ± 0.30	1.31 ± 0.20	0.820 ^a^	1.24 ± 0.10	1.20 ± 0.23	0.639 ^c^
ApoB/AI	0.82 ± 0.16	0.74 ± 0.17	0.312 ^a^	0.61 ± 0.13	0.65 ± 0.16	0.535 ^a^
IDL (mg/dL)	48.41 ± 26.34	43.50 ± 11.76	0.600 ^a^	28.53 ± 10.22	24.65 ± 12.91	0.466 ^a^
IDL-A (mg/dL)	10.00 ± 4.09	10.57 ± 4.70	0.853 ^a^	9.27 ± 5.25	7.06 ± 3.20	0.270 ^a^
IDL-B (mg/dL)	4.77 ± 4.35	5.76 ± 4.04	0.570 ^d^	2.75 ± 2.74	1.95 ± 2.18	0.479 ^a^
IDL-C (mg/dL)	33.64 ± 22.93	27.18 ± 7.97	0.418 ^a^	16.51 ± 9.32	10.46 ± 3.31	0.847 ^a^
LDL1 (mg/dL)	53.14 ± 23.95	39.76 ± 13.68	0.142 ^a^	37.49 ± 10.92	36.80 ± 11.31	0.891 ^a^
LDL2 (mg/dL)	30.87 ± 16.49	31.94 ± 15.01	0.881 ^a^	20.65 ± 7.37	22.89 ± 9.98	0.576 ^a^
LDL3 (mg/dL)	29.43 ± 7.59	19.36 ± 10.49	0.025 ^a^	15.65 ± 9.90	10.93 ± 4.55	0.248 ^d^
sdLDL (mg/dL)	32.08 ± 6.69	21.36 ± 12.31	0.030 ^c^	16.17 ± 10.69	12.36 ± 5.10	0.481 ^d^
L-HDL (mg/dL)	11.40 ± 9.03	10.81 ± 8.09	0.971 ^d^	10.35 ± 6.48	9.15 ± 6.78	0.690 ^a^
I-HDL (mg/dL)	21.11 ± 7.75	23.44 ± 9.66	0.560 ^a^	21.76 ± 5.75	20.94 ± 7.40	0.787 ^a^
S-HDL (mg/dL)	21.07 ± 8.51	22.04 ± 4.86	0.759 ^a^	20.90 ± 5.78	19.20 ± 3.75	0.447 ^a^
VLDL (mg/dL)	51.00 ± 9.56	47.80 ± 5.83	0.378 ^a^	35.5 ± 4.97	33.6 ± 6.85	0.487 ^a^
non-HDL (mmol/L)	4.92 ± 0.67	3.85 ± 0.44	0.001 ^c^	3.21 ± 0.57	2.88 ± 0.52	0.192 ^a^
TC/HDL-C ratio	181.13 ± 29.40	150.42 ± 38.81	0.061 ^a^	133.44 ± 29.81	135.95 ± 40.64	0.739 ^a^
oxLDL/HDL-C ratio	46.67 ± 13.04	38.72 ± 19.16	0.301 ^a^	37.86 ± 15.71	39.53 ± 15.75	0.815 ^a^
oxLDL/LDL-C ratio	14.95 ± 3.04	14.38 ± 2.08	0.645 ^a^	18.47 ± 3.21	18.96 ± 2.72	0.716 ^a^
oxLDL/TC ratio	9.93 ± 1.90	9.60 ± 2.03	0.719 ^a^	10.70 ± 2.37	11.02 ± 1.40	0.717 ^a^

TC, total cholesterol; TG, triglyceride; LDL-C, low-density lipoprotein cholesterol; HDL-C, high-density lipoprotein cholesterol; LPα, lipoprotein α; ApoB, apolipoprotein B; ApoAI, apolipoprotein AI; oxLDL, oxidized LDL; IDL, intermediate-density lipoprotein; LDL, low-density lipoprotein; sdLDL, small-density lipoprotein; HDL, high-density lipoprotein; L-HDL, large HDL; I-HDL, intermediate HDL;S-HDL, small HDL;VLDL, very-low-density lipoprotein. ^a^ Student *t* test; ^c^ Welch *t* tests; ^d^ Wilcoxon rank sum test. Data are presented as mean ± standard deviation (SD). Note: LDL4 was detected in 5, 4, 3, and 4 cases in POAG and control subjects in both elevated and normal TC and LDL-C level participants, respectively, while LDL5-LDL7 were not detected.

## Data Availability

Data is contained within the article and Supplementary Materials.

## Supplementary Materials

The supporting information can be downloaded at: https://www.mdpi.com/article/10.3390/ijms25084544/s1.
