# Supplementary material for: Profile of Lipoprotein Subclasses in Chinese Primary Open-Angle Glaucoma Patients
Source: Int J Mol Sci. 2024 Apr 21;25(8):4544. doi: 10.3390/ijms25084544 (PMC11050298; doi:10.3390/ijms25084544)
Supplement: Supplementary file 1 [file ijms-25-04544-s001.zip › ijms-2947671-supplementary.pdf]

Profile of Lipoprotein Subclasses in Chinese Primary Open-Angle Glaucoma Patients

Changzhen Fu [1], Jianming Xu [1], Shao-Lang Chen [1], Chong-Bo Chen [1], Jia-Jian Liang [1], Zibo Liu [1], Chukai Huang [1], Zhenggen Wu [1], Tsz Kin Ng [1,2], Mingzhi Zhang [1,*] and Qingping Liu [1,*]

1 Joint Shantou International Eye Center of Shantou University and The Chinese University of Hong Kong, Shantou 515041, China; fcz@jsiec.org (C.F.); xjm@jsiec.org (J.X.); csl@jsiec.org (S.-L.C.); ccb@jsiec.org (C.-B.C.); ljj@jsiec.org (J.-J.L.); liuzibo0910@gmail.com (Z.L.); hck@jsiec.org (C.H.); wzg@jsiec.org (Z.W.); micntk@hotmail.com (T.K.N.)
2 Department of Ophthalmology and Visual Sciences, The Chinese University of Hong Kong, Hong Kong, China
* Correspondence: zmz@jsiec.org (M.Z.); qingpingliu40@126.com (Q.L.)

Table S1. Lipid profiles in primary open-angle glaucoma patients

Variables	POAG (n = 20)			controls (n = 20)		
	with elevated TC and/or LDL-C (n = 10)	with normal TC and LDL-C (n = 10)	p Value	with elevated TC and/or LDL-C (n = 10)	with normal TC and LDL-C (n = 10)	p Value
TC (mmol/L)	6.31±0.88	4.58±0.42	<0.001[c]	5.30±0.40	4.16±0.67	<0.001[c]
LDL-C (mmol/L)	4.26±0.90	2.66±0.46	<0.001[c]	3.53±0.36	2.50±0.53	<0.001[a]
HDL-C (mmol/L)	1.39±0.33	1.37±0.25	0.909[a]	1.46±0.43	1.28±0.34	0.309[a]
TG (mmol/L)	2.08±0.89	1.39±0.64	0.065[a]	1.61±0.74	1.07±0.14	0.046[c]
oxLDL (U/L)	62.53±14.31	49.03±12.32	0.036[a]	50.05±9.22	46.89±9.79	0.480[a]
Lpα (mg/L)	133.90±93.34	141.20±127.4	0.885[a]	181.70±145.34	95.30±86.28	0.190[d]
ApoB (g/L)	1.10±0.22	0.75±0.16	0.001[a]	0.95±0.13	0.75±0.09	0.001[a]
ApoAI (g/L)	1.38±0.30	1.24±0.10	0.185[d]	1.31±0.20	1.20±0.23	0.242[a]
ApoB/AI	0.82±0.16	0.61±0.13	0.006[a]	0.74±0.17	0.65±0.16	0.261[a]
IDL (mg/dL)	48.41±26.34	28.53±10.22	0.047[c]	43.50±11.76	24.65±12.91	0.003[a]
IDL-A (mg/dL)	10.00±4.09	9.27±5.25	0.393[d]	10.57±4.70	7.06±3.20	0.067[a]
IDL-B (mg/dL)	4.77±4.35	2.75±2.74	0.231[a]	5.76±4.04	1.95±2.18	0.007[d]
IDL-C (mg/dL)	33.64±22.93	16.51±9.32	0.049[c]	27.18±7.97	10.46±3.31	0.013[a]
LDL1 (mg/dL)	53.14±23.95	37.49±10.92	0.076[a]	39.76±13.68	36.80±11.31	0.604[a]
LDL2 (mg/dL)	30.87±16.49	20.65±7.37	0.098[c]	31.94± 15.01	22.89±9.98	0.130[a]
LDL3 (mg/dL)	29.43±7.59	15.65±9.90	0.003[d]	19.36±10.49	10.93±4.55	0.043[d]
sdLDL (mg/dL)	32.08±6.69	16.17±10.69	0.001[d]	21.36±12.31	12.36±5.10	0.054[c]
L-HDL (mg/dL)	11.40±9.03	10.35±6.48	0.768[a]	10.81±8.09	9.15± 6.78	0.436[d]
I-HDL (mg/dL)	21.11±7.75	21.76±5.75	0.835[a]	23.44±9.66	20.94±7.40	0.524[a]
S-HDL (mg/dL)	21.07±8.51	20.90±5.78	0.959[a]	22.04±4.86	19.20±3.75	0.162[a]
VLDL (mg/dL)	51.00±9.56	35.5±4.97	<0.001[a]	47.80±5.83	33.6±6.85	<0.001[a]
non-HDL	4.92±0.67	3.21±0.57	<0.001[a]	3.85±0.44	2.88±0.52	<0.001[a]
TC/HDL-C ratio	181.13±29.40	133.44±29.81	0.002[a]	150.42±38.81	135.95±40.64	0.218[d]
oxLDL/HDL-C	46.67±13.04	37.86±15.71	0.189[a]	38.72±19.16	39.53±15.75	0.921[a]
oxLDL/LDL–C	14.95±3.04	18.47±3.21	0.021[a]	14.38±2.08	18.96±2.72	0.001[a]

oxLDL/TC ratio	9.93±1.90	10.70±2.37	0.436[a]	9.60±2.03	11.02±1.40	0.092[a]

TC, total cholesterol; TG, triglyceride; LDL-C, low-density lipoprotein cholesterol; HDL-C, high density lipoprotein cholesterol; Lpα, lipoprotein α; ApoB, apolipoprotein B; ApoAI, apolipoprotein AI; oxLDL, oxidized LDL; IDL, intermediate-density lipoprotein; sdLDL, small density lipoprotein; L-HDL, large HDL; I-HDL, intermediate HDL; S-HDL, small HDL;VLDL, very low-density lipoprotein; non-HDL, non-high-density lipoprotein cholesterol.

[a]Student *t* test, [c]Welch t tests; [d]Wilcoxon rank sum test.

Data are presented as mean ±SD.

Note: LDL4 was detected in 5, 4, 3, and 4 cases in POAG and control subjects in both elevated and normal TC and LDL-C level participants, respectively,while LDL5-LDL7 were not detected.
